# Human pluripotent stem cell-based organoids and cell platforms for modelling SARS-CoV-2 infection and drug discovery

**DOI:** 10.1016/j.scr.2021.102207

**Published:** 2021-05

**Authors:** Alice Maria Giani, Shuibing Chen

**Affiliations:** Department of Surgery, Weill Cornell Medicine, 1300 York Ave, New York, NY 10065, USA

**Keywords:** (6 MAX), SARS-CoV-2, COVID-19, Organoid, Stem cells, Personalized medicine, Drug discovery

## Abstract

The coronavirus disease 2019 (COVID-19) global pandemic caused by the novel severe acute respiratory syndrome coronavirus 2 (SARS-CoV-2) has affected over 200 countries and territories worldwide and resulted in more than 2.5 million deaths. In a pressing search for treatments and vaccines, research models based on human stem cells are emerging as crucial tools to investigate SARS-CoV-2 infection mechanisms and cellular responses across different tissues. Here, we provide an overview of the variety of human pluripotent stem cell-based platforms adopted in SARS-CoV-2 research, comprising monolayer cultures and organoids, which model the multitude of affected tissues *in vitro*. We highlight the strengths of these platforms, including their application to assess both the susceptible cell types and the pathogenesis of SARS-CoV-2. We describe their use to identify drug candidates for further investigation in addition to discussing their limitations in fully recapitulating COVID-19 pathophysiology. Overall, stem cell models are facilitating the understanding of SARS-CoV-2 and prove to be versatile platforms for studying infections.

## Highlights

• hPSC-derived cells/organoids are emerging tools to study the tropism of SARS-CoV-2 infection.
• hPSC-derived cells/organoids provide crucial tools to examine cellular response to SARS-CoV-2 infection.
• hPSC-derived cells/organoids can be applied to screen for drug candidates for COVID-19 patients.

## 1 Introduction

The number of confirmed COVID-19 cases worldwide has surpassed 100 million and is constantly growing. The majority of infected individuals experience only mild to moderate symptoms that do not require hospitalization (Wu and McGoogan, 2020) or are asymptomatic (Oran and Topol, 2020). The risk of developing severe infections increases with age and with the presence of preexisting medical conditions (Zhou et al., 2020a). Frequent symptoms in mild to moderately severe infections include fever, fatigue, and respiratory problems (Huang et al., 2020a). However, gastric symptoms (Jin et al., 2020, Pan et al., 2020), such as nausea or diarrhea, and neurological symptoms (Giacomelli et al., 2020, Nalleballe et al., 2020), such as headaches, loss of smell or taste, and confusion are not infrequent even in mildly symptomatic patients. Individuals that require hospitalization often develop respiratory deterioration resulting in pneumonia or even in acute respiratory distress syndrome, which is the most prevalent cause of death (Ruan et al., 2020). Many reports of severe damage to other organs, such as the cardiovascular system ( Shi et al., 2020b, Zheng et al., 2020), the gastrointestinal tract (Pan et al., 2020), the liver (Zhang et al., 2020b), the pancreas (Wang et al., 2020a), the kidneys (Pei et al., 2020a) and the nervous system (Mao et al., 2020), indicate that SARS-CoV-2 infection might cause serious, or even lethal, injuries in organs other than the respiratory system. Indeed, several deaths have been documented due to heart failure (Guo et al., 2020), renal failure (Cheng et al., 2020) or multi-organ failure (Chen et al., 2020).

The experimental tools currently available to investigate SARS-CoV-2 biology and COVID-19 pathophysiology include human biopsy samples, animal models, animal cell lines and different types of human cell and organoid platforms. Human biopsies are a very useful resource to understand the pathology of COVID-19 and to assess the validity and relevance of other model systems. However, biopsies are limited as a broader applied research tool due to the paucity of samples available and the short time they can be maintained ex vivo. So, a profusion of animal models has been used in COVID-19 studies, ranging from small animals, including transgenic mice expressing human ACE2 and syrian hamsters, to larger animals, such as ferrets, cats and non-human primates (Bao et al., 2020, Chan et al., 2020, Jiang et al., 2020, Kim et al., 2020b, Munster et al., 2020, Rockx et al., 2020, Shi et al., 2020a). Animal-derived cells have also been extensively used to amplify and isolate SARS-CoV-2, investigate infection mechanisms and perform drug screening studies. So far, the majority of studies using non-human cell lines have relied on Vero cells (Harcourt et al., 2020, Matsuyama et al., 2020, Wang et al., 2020b, Zhou et al., 2020c), kidney epithelial cells isolated from an African green monkey. However, due to their evolutionary distance from humans, animal models and animal-derived cells cannot fully recapitulate characteristic features of human physiology and diseases. To address this limitation, several human cell lines have been used to study SARS-CoV-2 biology, including immortalized cell lines, cancer cell lines, and differentiated stem cells. Among immortalized and cancer human cell lines, Calu-3 and A549 (lung adenocarcinoma), Caco-2 (colorectal adenocarcinoma), HFL and MRC-5 (fetal lung fibroblasts), HEK293T (embryonic kidney), Huh7 (hepatocellular carcinoma), HeLa (cervical cancer), U251 (glioblastoma) and RD (rhabdomyosarcoma) have been widely employed, observing distinct susceptibilities to SARS-CoV-2 infection and viral replication rates in different cell types (Chu et al., 2020, Harcourt et al., 2020, Hoffmann et al., 2020, Kim et al., 2020a, Ou et al., 2020, Riva et al., 2020, Shang et al., 2020, Wang et al., 2020b). Immortalized and cancer human cell lines have been useful to study some aspects of SARS-CoV-2 infection and replication. However, they fail to recapitulate *in vitro* the diversity of cell types present in human organs. These cell lines also generally carry cancer-associated mutations in genes controlling cell cycle and proliferation (Blanco et al., 2009) and can have mutations in genes regulating the innate immune response (Hare et al., 2016). Therefore, immortalized and human cancer cell lines are limited in their ability to accurately model the cell type-specific susceptibility and response to SARS-CoV-2 infection.

Human pluripotent stem cell (hPSC) have rapidly emerged as an alternative to animal models as well as to immortalized and cancer human cell lines, since they are human cells that have the ability to self-renew indefinitely and differentiate into cells of the three germ layers. They avoid interspecies differences and can be used to obtain abundant samples of a variety of different cell types. Under precise differentiation conditions, hPSCs, including embryonic stem cells (hESCs) and induced pluripotent stem cells (iPSCs), can generate specific cell types in monolayer cultures. In addition, over the last few years numerous differentiation protocols have been developed to generate three-dimensional (3D) cultures, known as organoids, which more faithfully recapitulate human organs *in vitro*.

**Fig. 1:**
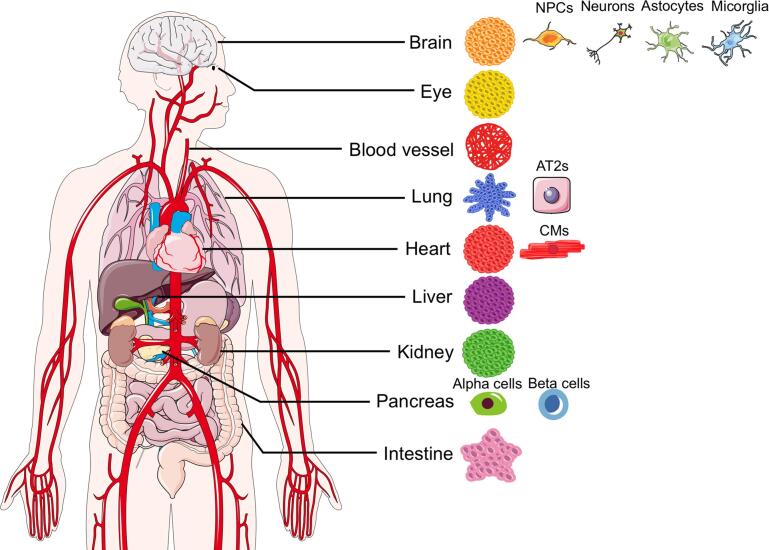
hPSC-based Models of SARS-CoV-2 Infection. Schematic representation of the hPSC-based monolayer cultures and organoids used to date to study SARS-CoV-2 tropism and COVID-19 pathophysiology across different organs.

Both hPSC-derived monolayer cultures and organoids have already been used to investigate host-virus interactions in different human cell types and tissues, including modelling respiratory infections, such as influenza, enteric infections, such as those due to norovirus and rotavirus, hepatic infections, such as hepatitis B and hepatitis C, infections of components of the immune systems, as in HIV and dengue virus studies, and both prenatal and postnatal brain infections, including those caused by Zika virus and herpes simplex virus 1. In the case of Zika virus, they have also been employed to identify antiviral drug candidates (Xu et al., 2016, Zhou et al., 2017). Several of these hPSC-based platforms have been adapted to study SARS-CoV-2 biology and COVID-19 pathophysiology (Fig. 1 and Table 1). In this review, we describe how they are used to determine SARS-CoV-2 tropism, investigate infection mechanisms and identify potential treatments in different organs, highlighting their strengths compared to other model systems. We also address their limitations in fully recapitulating COVID-19 pathophysiology, while proposing potential improvements and new applications.

**Table 1:** Summary of hPSC-derived cells and organoids used to study SARS-CoV-2 infection.

**Organ**	**hPSC-based model**	**Cell Culture Method**	**MOI**	**Timepoints analyzed**	**Permissiveness to SARS-CoV2**	**Pathological Features**	**Drug screening: Candidate Hits**	**Reference**
Lung	alveolospheres	3D	MOI = 0.1, 0.5	24 hpi	yes	not described in detail	not reported	Dobrindt et al., 2020

Lung	alveolar organoids	3D	MOI = 0.01 for 24 h	24 hpi	yes	proinflammatory cytokine response	imatinib, mycophenolic acid (MPA), quinacrine dihydrochlorid (QNHC)	Han et al., 2020
Lung	airway organoids, alveolar organoids	3D	MOI = 1 for 2 h	2, 24, 48, 72, 96 hpi	yes, ciliated cells, club cellscells and a subpopulation of alveolar type 2 cells	decreased expression of genes relatred to metabolic processes; , proinflammatory cytokine response; activation of NF-kB-mediated inflammatory signalling; cell death; downregulation of ACE2 mRNA	remdesivir, to a less extent camostat only in airway organoids ari a	Pei et al., 2020b

Lung	alveolar organoids	3D	MOI = 0.1 for 2 h	72 hpi	yes	not described in detail	drugs targeting androgen signaling such as dutasteride, ketoconazole and finasteride	Samuel et al., 2020
Lung	alveolar epithelial type 2 cells, iAT2 alveolospheres	2D/3D	MOI = 0.0004, 0.004, 0.4, 0.5, 2.5, 5 for 1 h	1, 2, 4 dpi	yes	decreased expression of AT2-specific genes; activation of NF-kB-mediated inflammatory signalling; limited and delayed induction of IFN signalling; cell death	remdesivir, camostat	Huang et al., 2020b

Lung	alveolar epithelial type 2 cells	2D/3D	MOI = 10 for 1 h	2 dpi	yes	not described in detail	lactoferrin, lactoferrin + remdesivir	Mirabelli et al., 2020

Lung	pneumocyte-like cells	2D	1 × 10^5^ PFU	2 dpi	yes	not described in detail	ONO-5334, MDL28170, apilimod	Riva et al., 2020
**Cardiovascular system**								

Heart	vascular organoids	3D	10^2^, 10^4^, or 10^6^ infectious particles for 1 h	3 and 6 dpi	yes	not described in detail	clinical grade human recombinant soluble ACE2 (hrsACE2)	Monteil et al., 2020
Heart	cardiomyocytes	2D	MOI = 0.01, 1 for 2 h	24, 48, 72, 96 hpi	yes	proinflammatory cytokine response; cell death; impaired contractility; upregulation of genes related to reactive oxygen stress	clinical grade human recombinant soluble ACE2, N-acetyl-L-leucyl-L-leucyl-Lmethionine (ALLM), remdesivir	Bojkova et al., 2020

cardiospheres	3D	25 uL of 1 × 10^7^ TCID 50/mL for 3–5 days	3 and 5 dpi

Heart	cardiomyocytes	2D	MOI = 0.01, 0.1 for 1 h	24, 48, 72 hpi	yes	cell death; impaired contractility	berzosertib	Garcia et al., 2020
Heart	cardiomyocytes	2D	MOI = 0.1, 5 for 1 h	12, 24, 36, 48, 60, 72, 96, 120 hpi	yes	cell death; impaired contractility; altered electrophysiological properties; innate immune response activation	not reported	Marchiano et al., 2020

engineered heart tissues	3D	MOI = 10 for 1 h	24, 48, 72, 96, 120,144, hpi
Heart	cardiomyocytes	2D	MOI = 0.01	3, 7 dpi	yes	cell death; sarcomere disorganization	bromodomain protein 4 inhibitors as INCB054329	Mills et al., 2020

cardiac organoids	3D	MOI = 0.01

Heart	cardiomyocytes, cardiac fibroblasts, endothelial cells	2D	MOI = 0.001, 0.006, 0.01, 0.1	24, 48, 72 hpi	yes, cardiomyocytes	cell death; myofibrillar fragmentation; abnormal nuclear structure; innate immune response activation	apilimod, bafilomycin, E64d, Z-Phe-Tyr(tBu)-diazomethylketone (Z-FY-DK)	Pérez-Bermejo et al., 2020
Heart	cardiomyocytes	2D	not reported	not reported	not reported	not reported	drugs targeting androgen signaling such as dutasteride, ketoconazole and finasteride	Samuel et al., 2020

Heart	cardiomyocytes	2D	MOI = 0.1 for 72 h	72 hpi	yes	proinflammatory cytokine response; cell death; impaired contractility; downregulation of transcriptional pathways related to mitochondria function and oxidative phosphorylation	not reported	Sharma et al., 2020

Heart	cardiomyocytes	2D	pseudovirus MOI = 0.01 for 2 h	24 hpi	yes	not described in detail	not reported	Yang et al., 2020

**Kideny**								

Kidney	kidney organoids	3D	10^3^ or 10^5^ infectious particles for 1 h	6 dpi	yes	not described in detail	clinical grade human recombinant soluble ACE2 (hrsACE2)	Monteil et al., 2020
**Gastrointestinal Tract**								

Intestine	intestinal organoids	3D	MOI = 0.05, 0.5, 1 for 24 h and 48 h	24 and 48 hpi	yes	not described in detail	not reported	Dobrindt et al., 2020

Colon	colonic organoids	3D	MOI = 0.1 for 24 h	24 hpi	yes, all cell types	proinflammatory cytokine response; increased expression of genes involved in oxidative phosphorylation, production of reactive oxygen species and nitric oxide; cell death	imatinib, mycophenolic acid (MPA), quinacrine dihydrochlorid (QNHC)	Han et al., 2020
Intestine	intestinal organoids	3D	3 × 10^5^ PFU for 1 h	24 and 48 hpi	yes, enterocytes, enteroendocrine cells, Paneth cells	cell death	remdesivir, EK1	Krüger et al., 2020

**Liver**								

Liver	liver organoids	3D	pseudovirus MOI = 0.01 for 2 h	24 hpi	yes, mainly albumine-positive hepatocytes	not described in detail	not reported	Yang et al., 2020

**Pancreas**								

Pancreas	pancreatic endocrine cultures	2D	MOI = 0.01, 0.05, 0.1 for 24 h	24 hpi	yes, mainly alpha and beta cells	proinflammatory cytokine response; cell death of alpha and beta cells	not reported	Yang et al., 2020
**Brain**								

Brain	brainspheres	3D	MOI = 0.1 for 6 h	6, 72 hpi	yes, few neurons	not described in detail	not reported	Bullen et al., 2020
Brain	glutamatergic neurons	2D	MOI = 0.05, 0.2, 1	24, 48 hpi	yes	not described in detail	not reported	Dobrindt et al., 2020

Brain	cortical neurons, astrocytes, microglia	2D	MOI = 0.2, 1, 5 for 12 h	24, 48, 120 hpi	yes, mainly choroid plexus epithelial cells	proinflammatory cytokine response; cell death of both infected and uninfected cells; formation of syncytia; increased expression of genes reated to RNA processing, cytoskeletal rearrangement and vascular remodeling; functional deficits of ChP cells; damage of ChP organoids integrity	not reported	Jacob et al., 2020

cortical, hippocampal, hypothalamic, midbrain and chorpid plexus, organoids	3D	10^3^, 10^4^, or 10^5^ focus forming units for 8 h (estimated MOI = 0.1–0.05)	24, 72 hpi

Brain	cortical organoids	3D	MOI = 2.5 for 3 days	7 dpi	yes, neural progenitor cells and cortical neurons	reduced number of excitatory synapses; cell death	sofosbuvir	Mesci et al., 2020
Brain	cerebral organoids ,choroid plexus organoids	3D	expected MOI = 0.5, 5	24, 48, 72, 96 hpi	yes, mainly choroid plexus epithelial cells	damage of tight-junctions and integrity of choroid plexus organoids	not reported	Pellegrini et al., 2020

Brain	brain organoids, organotypic slices	3D	MOI = 1.8 × 10^–4^, 8.8 × 10^–5^	2, 4, 6 dpi	yes, mainly neruons	dysregulated localization of Tau protein; hyperphosphorilation of Tau protein; cell death	not reported	Ramani et al., 2020

Brain	neural progenitor cells	2D	not available	6, 12, 48 hpi	yes, neural progenitor cells and cortical neurons	cell death of infected and neighbouring cells; increased expression of genes related to cell division, organelle fission and metabolic processes; diverging metabolic changes in infected versus neighboring cells	not reported	Song et al., 2020

brain organoids	3D	MOI = 1	2, 24, 96 hpi

Brain	cortical neurons, dopaminergic neurons, microglia	2D	pseudovirus MOI = 0.01 for 2 h	24 hpi	yes, mainly dopaminergic neruons	not described in detail	not reported	Yang et al., 2020

Brain	neural progenitor cells	2D	MOI = 10	24, 48 hpi	yes, neural progenitor cells and cortical neurons	cell–cell fusion; cell death	not reported	Zhang et al., 2020a

neurospheres, brain organoids	3D	6 × 10^6^ PFU/mL for 24 h	24, 48, 72 hpi
**Eye**								

Eye	whole-eye SEAM organoids	3D	MOI = 1 for 24 h	24 hpi	yes, mainly corenal cells	proinflammatory cytokine response; altered experssion of cell cycle-related genes	not reported	Makovoz et al., 2020

## 2 hPSC-based platforms to study SARS-CoV-2 infection

**Fig. 2:**
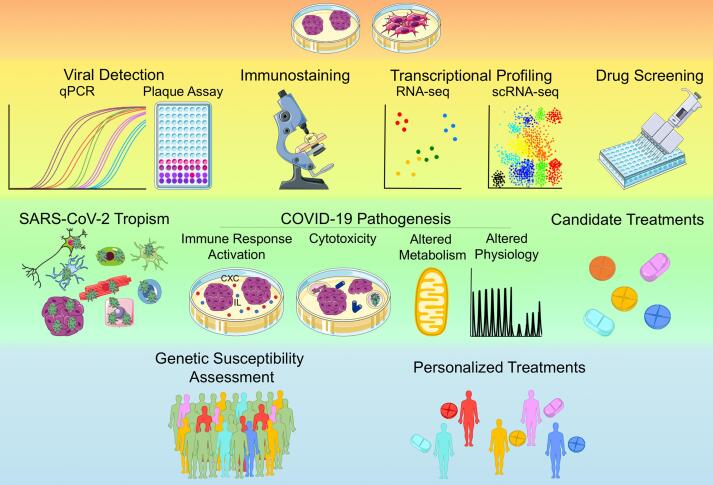
Applications of hPSC-based Models in COVID-19 research. Schematic illustration of how hPSC-based platforms are used to investigate the cell-type-specific susceptibility and response to SARS-CoV-2 infection as well as to identify new candidate treatments. Assays and applications are placed on different backgrounds. Yellow: common assays performed to date using hPSC-based platforms, Green: currently widespread applications of hPSC-based platforms, Light blue: potential future applications of hPSC-based platforms. (For interpretation of the references to colour in this figure legend, the reader is referred to the web version of this article.)

COVID-19 studies using hPSC-derived monolayer cultures and organoids have often employed similar approaches and observed common patterns, even in different cell types and tissues (Fig. 2). In terms of approaches, a widely adopted strategy to identify the cell types potentially susceptible to the virus has been to monitor the expression profiles of SARS-CoV-2 entry receptor angiotensin-converting enzyme 2 (ACE2). Many studies have also examined the expression of serine protease TMPRSS2, which cleaves SARS-CoV-2 Spike protein at two sites enabling the fusion of the cellular and viral membranes, and other putative priming proteases, such as Furin, TMPRSS4 and TMPRSS11E. The expression profiles of these key mediators of SARS-CoV-2 infection identified *in vitro* have usually been compared to those in primary human tissues, confirming hPSC-derived cells and organoids as reliable models. Indeed, hPSC-derived cells expressing ACE2 and TMPRSS2 or other putative entry receptors and priming proteases become infected with SARS-CoV-2. A common pattern that has been observed across models of different tissues is the increased expression of genes involved in the innate immune response, as chemokines, interleukins and other cytokines upon SARS-CoV-2 infection (Fig. 2 and Table 1). Another shared transcriptional signature is the reduced expression of genes related to metabolic activity and cell function, which is frequently accompanied by a time-dependent upregulation of apoptotic genes (Fig. 2 and Table 1). Increased cell death after infection has indeed been confirmed from protein expression and cell counts. However, whether infected or neighboring cells are the most affected by cell death seems to depend on the tissue examined. Changes in cell physiology after infection have also been reported (Fig. 2 and Table 1). Aside from these general approaches and patterns, organ-specific signatures have been described, which we review in the following sections. Overall, additional studies are still required to further assess the clinical relevance of these *in vitro* findings.

### 2.1 Lung

The lungs are the major target of SARS-CoV-2 and infected individuals frequently present with respiratory symptoms (Huang et al., 2020a). HPSC-derived airway (hAWOs) (Pei et al., 2020b) and alveolar (hALOs) organoids (Dobrindt et al., 2020, Han et al., 2020, Huang et al., 2020b, Pei et al., 2020b, Samuel et al., 2020) as well as monolayer cultures of alveolar epithelial type 2 cells (hAT2) (Huang et al., 2020b) have been used to investigate SARS-CoV-2 tropism and the early phases of infection in the lungs. Independent studies using hPSC-derived lung organoids observed that ACE2 is mainly expressed in ciliated cells and in a subpopulation of hAT2 cells, while TMPRSS2 is expressed in the majority of cells (Dobrindt et al., 2020, Han et al., 2020, Pei et al., 2020b), in agreement with their expression in adult human lungs (Hou et al., 2020). Analysis of ACE2 expression in monolayer cultures of hAT2 produced similar findings (Huang et al., 2020b). Upon viral exposure, ciliated cells, club cells and a subpopulation of hAT2 cells become infected, while alveolar type 1 (AT1) cells, basal cells, goblet cells, proliferating cells and pulmonary neuroendocrine cells have few or no signs of infection in hPSC-derived lung organoids. These findings are consistent with data from lung autopsies of COVID-19 patients and primary lung airway organoids, air–liquid interface (ALI) cultures and AT2 alveolar organoids derived from lung biopsies (Katsura et al., 2020, Lamers et al., 2020, Purkayastha et al., 2020, Youk et al., 2020). The susceptibility to infection of specific pulmonary cell populations was also confirmed in monolayer cultures (Huang et al., 2020b, Mirabelli et al., 2020, Riva et al., 2020). Transcriptional profiling of infected hPSC-derived lung organoids and hAT2 cultures revealed an increased expression of genes associated with the activation of the immune response, as cytokines, chemokines, members of TNF signaling, IL-17 signaling and the NF-kB family (Han et al., 2020, Huang et al., 2020b, Pei et al., 2020b). On the other hand, genes associated with lipid metabolism were downregulated, along with the expression of ACE2 and TMPRSS2. A delayed and moderate activation of genes related to IFN signaling but not type I and III IFN genes was reported in hAT2 cultures four days post infection along with the progressive downregulation of hAT2 specific genes and upregulation of apoptotic genes (Huang et al., 2020b). A progressive increase in cell death, becoming evident from three days post infection, was further confirmed by immunostaining in lung organoids (Pei et al., 2020b) and hAT2 cultures (Huang et al., 2020b).

### 2.2 Cardiovascular system

As cardiovascular complications are another common feature of severe COVID-19 patients (Zheng et al., 2020), hPSC-derived vascular organoids (Monteil et al., 2020), cardiac organoids (Bojkova et al., 2020, Marchiano et al., 2020, Mills et al., 2020) and monolayer cultures of cardiomyocytes (hPSC-CMs) (Bojkova et al., 2020, Garcia et al., 2020, Marchiano et al., 2020, Mills et al., 2020, Pérez-Bermejo et al., 2020, Samuel et al., 2020, Sharma et al., 2020, Yang et al., 2020) have been explored as potential *in vitro* models to study SARS-CoV-2 infection in the cardiovascular system. High levels of cardiac expression of ACE2 have been detected in pericytes lining blood vessels as well as in human cardiomyocytes, endothelial cells and vascular smooth muscle cells (Nicin et al., 2020, Tucker et al. , 2020). Consistent with these findings, immunostainings, bulk RNA-seq and single cell RNA-Seq (scRNA-seq) data have confirmed increasing levels of ACE2 expression in hPSCs-CMs with differentiation progression (Bojkova et al., 2020, Marchiano et al., 2020). To investigate whether SARS-CoV-2 can infect human blood vessels, Monteil et al. have exposed hPSCs-derived capillary organoids to the virus. SARS-CoV-2 was able to enter into vascular organoids composed of haematopoietic cells, CD31 + endothelial cells, pericytes and mesenchymal stem-like cells (Monteil et al., 2020). HiPSC-CMs and cardiac organoids have also been found susceptible to SARS-CoV-2 pseudovirus and SARS-CoV-2 virus infection (Bojkova et al., 2020, Garcia et al., 2020, Marchiano et al., 2020, Mills et al., 2020, Pérez-Bermejo et al., 2020, Sharma et al., 2020, Yang et al., 2020). Upon exposure to SARS-CoV-2, the proportion of apoptotic hPSCs-CMs significantly increases, their electrophysiological properties and sarcomere organization is altered, and contractility of both hPSC-CM and cardiac organoids is reduced (Marchiano et al., 2020, Mills et al., 2020, Pérez-Bermejo et al., 2020, Sharma et al., 2020). Transcriptional profiling of infected hPSCs-CMs revealed increased expression of genes involved in apoptosis and in the innate immune response, as cytokines and interleukins, while genes involved in oxidative phosphorylation, mitochondrial and cardiac function were downregulated (Sharma et al., 2020). These transcriptional alterations suggest that SARS-CoV-2 infection of hPSCs-CMs results in changes in cellular metabolism, activation of the inflammatory response and increased cell death.

### 2.3 Kidney

Given the presence of renal complications in COVID-19 patients (Kunutsor and Laukkanen, 2020) and the detection of SARS-CoV-2 in the urine of infected individuals (Ling et al., 2020), hPSC-derived kidney organoids have also been used as a model of SARS-CoV-2 infection. Consistent with ACE2 expression profiles in human kidney biopsies, ACE2 is expressed in proximal tubular cells and podocytes, but not in mesenchymal, renal endothelial-like or proliferating cells in human kidney organoids. Exposure to SARS-CoV-2 resulted in infection of kidney organoids (Monteil et al., 2020).

### 2.4 Gastrointestinal tract

Colonic (hPSC-COs) and intestinal (PSC-HIOs) organoids derived from hPSCs have been used to investigate the cell type-specific susceptibility to SARS-CoV-2 infection in the gastrointestinal tract (Dobrindt et al., 2020, Han et al., 2020, Krüger et al., 2020). Transcriptional profiling of hPSC-COs indicates that ACE2 and TMPRSS2 are highly expressed in enterocytes and at lower levels in all the other cell types present in these organoids, including goblet cells, neuroendocrine cells, transit amplifying cells and stem cells (Han et al., 2020). Expression of ACE2 and TMPRSS2 has also been confirmed by immunostaining in the majority of cell types composing PSC-HIOs, including enterocytes, enteroendocrine cells and Paneth cells, with the exception of goblet cells (Krüger et al., 2020). Exposure to a SARS-CoV-2 pseudovirus tagged with luciferase infected all cell types present in hPSC-COs cultured *in vitro* and transplanted into mice, with ACE2-positive cells and enterocytes being the most affected cell types (Han et al., 2020). Colonic and intestinal organoids cultured *in vitro* were also susceptible to infection by SARS-CoV-2 virus (Dobrindt et al., 2020, Han et al., 2020, Krüger et al., 2020), in agreement with data of primary intestinal organoids derived from human biopsies (Lamers et al., 2020, Zang et al., 2020, Zhou et al., 2020b). Differential gene expression analysis identified an increased expression of chemokines and other cytokine genes, transcripts related to the production of reactive oxygen species and nitric oxide, and genes involved in oxidative phosphorylation in infected hPSC-COs (Han et al., 2020). Increased cell death post-infection was observed in both hPSC-COs and PSC-HIOs (Han et al., 2020, Krüger et al., 2020).

### 2.5 Liver

As hepatic dysfunction has also been observed in COVID-19 patients (Zhang et al., 2020b), hPSCs-derived liver organoids have been tested as models of SARS-CoV-2 infection. HPSC-derived liver organoids composed mainly of albumin-positive hepatocytes expressed ACE2 in the majority of cells and were permissive to SARS-CoV-2 pseudovirus infection (Yang et al., 2020), in line with findings in primary hepatocyte and ductal organoids derived from adult biopsies (Zhao et al., 2020, Yang et al., 2020).

### 2.6 Pancreas

In hPSC-derived pancreatic endocrine cultures, alpha and beta cells but not delta cells stained positive for ACE2 and were permissive to SARS-CoV-2 pseudovirus infection, both when cultured *in vitro* or transplanted in mice (Yang et al., 2020). These findings are in agreement with the reported expression profiles of key mediators of SARS-CoV-2 infection and susceptibility to viral entry of adult human pancreatic islets (Yang et al., 2020). Transcriptional analysis of SARS-CoV-2 infected hPSC-derived pancreatic endocrine cultures suggested an increased death rate of alpha and beta cells after infection, as chemokine genes and genes involved in the insulin resistance pathway were upregulated in infected samples while genes associated with metabolic activity, glucagon signaling, calcium signaling, and other pathways related to alpha and beta cells showed reduced expression. The increased cell death of alpha and beta cells after infection has been confirmed by immunostaining, further supporting that these cells are also targets of SARS-CoV-2 (Yang et al., 2020).

### 2.7 Brain

COVID-19 patients frequently present with neurological symptoms (Helms et al., 2020, Mao et al., 2020). The question whether these neurological manifestations are due to direct SARS-CoV-2 infection or to secondary damage of the nervous system has been extensively investigated since the beginning of the pandemic. To date, SARS-CoV-2 RNA transcripts have been detected in human brain autopsies (Paniz-Mondolfi et al., 2020, Puelles et al., 2020) and in the cerebrospinal fluid (Moriguchi et al., 2020, Virhammar et al., 2020) of few COVID-19 patients. However, several clinical studies reported mixed findings or did not detect SARS-CoV-2 presence in brain tissues (Schaller et al., 2020, Solomon et al., 2020). To experimentally investigate if SARS-CoV-2 can directly infect cells of the human brain and what are the effects of infection, monolayer cultures of hPSCs-derived neural progenitor cells (hNPCs), neurons, astrocytes and microglia as well as three-dimensional neurospheres and brain organoids have been used (Bullen et al., 2020, Dobrindt et al., 2020, Jacob et al., 2020, Mesci et al., 2020, Pellegrini et al., 2020, Ramani et al., 2020, Song et al., 2020, Yang et al., 2020, Zhang et al., 2020a). Neurospheres are 3D organoids composed of hNPCs that mimic *in vitro* the early stages of neurogenesis, while brain organoids model different brain regions at later stages of human neurodevelopment and are composed of hNPCs and more differentiated cells of the neuronal lineage, such as post-mitotic neurons and astrocytes. Independent studies using both hPSC-derived monolayer cultures and organoids have confirmed ACE2 expression at low to moderate levels in hPSC-derived cortical neurons, at low to moderate levels in astocytes and hNPCs both grown in monolayer cultures and organoids, and at higher levels in dopaminergic neurons and choroid plexus (ChP) organoids (Bullen et al., 2020, Dobrindt et al., 2020, Jacob et al., 2020, Mesci et al., 2020, Pellegrini et al., 2020, Ramani et al., 2020, Song et al., 2020, Yang et al., 2020; (Zhang et al., 2020a)). SARS-CoV-2 pseudovirus robustly infected monolayer cultures of dopaminergic neurons and ChP organoids while few microglia and cortical neurons become infected (Pellegrini et al., 2020, Yang et al., 2020). Upon SARS-CoV-2 exposure, viral entry was observed in hNPCs and cortical neurons both grown in monolayer cultures or organoids, as indicated by immunostaining. However, the reported percentages of infected cortical neuronal cells vary across studies (Table 1). Also the efficiency of viral replication in hPSC-based cortical models appears controversial, with some studies reporting increased levels of viral RNA in infected cerebral organoids and their supernatants (Bullen et al., 2020, Zhang et al., 2020a), while others did not observe changes in the number of infected cells and in the levels of viral RNA in the supernatant detected at two and four days post infection (Ramani et al., 2020). These conflicting findings are likely due to differences in experimental conditions across studies, such as the MOI used, the timepoints examined and the adoption of hPSC-based models at different stages of differentiation (Table 1). Consensus is found that a subpopulation of ChP epithelial cells expressing ACE2 is particularly susceptible to SARS-CoV-2 infection and permissive to viral replication (Jacob et al., 2020, Pellegrini et al., 2020). After SARS-CoV-2 exposure, infected cells tended to form syncytia and the tight junctions between ChP epithelial cells became progressively disrupted resulting in loss of integrity of the blood-cerebrospinal fluid barrier (Jacob et al., 2020, Pellegrini et al., 2020), that might enable the entry of SARS-CoV-2 as well as immune cells and proinflammatory cytokines in the brain. Astrocytes and neurons in hippocampal, hypothalamic, and midbrain organoids were also susceptible to SARS-CoV-2 infection (Jacob et al., 2020). Increased cell death was observed in both infected and non-infected hNPCs, astrocytes and cortical neurons (Mesci et al., 2020, Ramani et al., 2020, Song et al., 2020, Zhang et al., 2020a) as well as in ChP epithelial cells (Jacob et al., 2020, Pellegrini et al., 2020), starting from three days post infection. Other reported consequences of infection were a reduction in the number of excitatory synapses in cortical neurons (Mesci et al., 2020), dysregulated localization and increased phosphorylation of Tau protein (Ramani et al., 2020), and transcriptional alterations indicating activation of proinflammatory cellular responses and metabolic processes (Jacob et al., 2020, Song et al., 2020).

### 2.8 Eye

SARS-CoV-2 tropism and mechanisms of infection have also been investigated using hPSC-derived whole-eye SEAM organoids, comprising cells of the cornea, iris, ciliary margin, lens, retina and retinal pigment epithelium (Makovoz et al., 2020). ACE2 was expressed in a large number of corneal cells, with a subset also coexpressing TMPRSS2 (Makovoz et al., 2020). Corneal cells with elevated expression of ACE2 also expressed high levels of other putative SARS-CoV-2 entry genes, such as TMPRSS11E, BSG (Basigin) and FURIN (Makovoz et al., 2020). Consistent with the expression of ACE2 and TMPRSS2 in subsets of corneal cells, especially in limbal cells, hPSCs-derived eye organoids were susceptible to SARS-CoV-2 infection. Expression of genes related to cell cycle and proinflammatory cytokine response, especially mediated by NFκB, were increased in infected samples. Similar findings were obtained from SARS-CoV-2 infection of human ocular biopsies, supporting the utility of using hPSCs-derived whole-eye organoids to study SARS-CoV-2 infection and test candidate drugs (Makovoz et al., 2020).

## 3 hPSC-based platforms as tools to identify COVID-19 treatments

The identification of drugs to treat COVID-19 and prevent SARS-CoV-2 infection is paramount in the response to the pandemic. Since the development of novel drugs can take 10 or more years (Van Norman, 2016), the repurposing of existing drugs with known pharmacokinetic and safety profiles is a rapid, attractive alternative. Several COVID-19 clinical trials and *in silico* or *in vitro* screenings of drugs that are commercially available, currently in clinical trials for other pathologies, or already characterized in preclinical studies, have already begun (Riva et al., 2020, Wu et al., 2020, Zhou et al., 2020d). These drugs either target SARS-CoV-2 directly, or act on human cells and the immune system. In this context, hPSC-based platforms are used to validate the efficacy of selected candidate drugs, or to identify compounds to repurpose through high-throughput screening of chemical libraries (Fig. 2). In addition to the potential clinical applications, analysis of the pathways modulated by the identified hit drugs can also improve the knowledge about COVID-19 pathophysiology.

### 3.1 Lung

Reflecting the prevalence of respiratory symptoms, several studies are using hPSCs-derived lung monolayer cultures and organoids to identify drugs for the treatment of COVID-19 (Han et al., 2020, Huang et al., 2020b, Mirabelli et al., 2020, Pei et al., 2020b, Riva et al., 2020, Samuel et al., 2020). The majority of studies are using hPSCs-derived lung cells and organoids to confirm the antiviral activity of candidate drugs identified based on literature review or through large-scale screenings using animal-derived cells, immortalized and cancer human cell lines. For instance, hiPSC-derived alveolar epithelial type 2 cells (hiAT2) have been used to confirm the antiviral activity of three promising drugs, including camostat, remdesivir, and E-64d (Huang et al., 2020b). Both camostat and remdesivir treatment successfully reduced the presence of viral transcripts in hiAT2s, further confirming the efficacy of these compounds *in vitro*. On the other hand, administration of the cathepsin B and L inhibitor E-64d was ineffective in hiAT2s. Adopting the same literature-based strategy for selecting candidate drugs, Pei et al. tested the antiviral activity of camostat, remdesivir, bestatin and neutralizing antibody CB6 in hESC-derived airway (hAWOs) and alveolar (hALOs) organoids, observing similar results (Pei et al., 2020b). Remdesivir was the most effective drug, as it significantly reduced the production of infectious virus and viral load in both hAWOs and hALOs, while camostat partially reduced the production of infectious virus only in hAWOs, and bestatin was ineffective in both types of lung organoids. Also neutralizing antibody CB6 significantly reduced the production of infectious viral particles in lung organoids (Pei et al., 2020b). Human iPSC-derived pneumocytes have also been used to confirm the antiviral activity of three drugs identified in a large-scale screening study using infected Vero E6 cells (Riva et al., 2020), including the cathepsin K inhibitor ONO-5334, the calpain and cathepsin B inhibitor MDL28170, and the PIKfyve kinase inhibitor apilimod that is used to treat autoimmune diseases and has also anticancer and antiviral properties. From another drug screening study that tested the antiviral activity of 1441 compounds in Vero E6 cells, administration of lactoferrin alone or in combination with other drugs such as remdesivir emerged as the most promising candidate treatments for further investigation (Mirabelli et al., 2020). These drugs inhibited SARS-CoV-2 infection in a dose-dependent manner in iPSC-derived alveolar epithelial type 2 cells (iAEC2s), confirming their suitability for further clinical studies. Human lung organoids (HLOs) have also been used to confirm the efficacy in reducing SARS-CoV-2 infection of antiandrogenic compounds, such as dutasteride, ketoconazole and finasteride, that had been identified as hit drugs from a combination of *in vitro* and *in silico* screenings (Samuel et al., 2020). Additionally, in a study from our group, hAWOs were adapted to a high-throughput screening platform (Han et al., 2020). In this study, hAWOs infected with a SARS-CoV-2 pseudovirus were treated with the Prestwick chemical library, containing 1,280 approved drugs selected for their high chemical and pharmacological diversity. The screening identified three FDA-approved lead drugs, including imatinib, mycophenolic acid (MPA), and quinacrine dihydrochloride (QNHC) that were further investigated (Han et al., 2020). Imatinib is an inhibitor of several tyrosine kinases used as an anticancer medication, and it is also able to inhibit *in vitro* the replication of SARS-CoV and MERS-CoV (Coleman et al., 2016). MPA is an immunosuppressant drug used for autoimmune diseases and to avoid organ rejection, and it is also able to inhibit the replication of several viruses (Chapuis et al., 2000, Cheng et al., 2015, Diamond et al., 2002). QNHC is an anti-malarial drug that has been used to treat intestinal infections and autoimmune diseases (Toubi et al., 2006). Treatment with imatinib, MPA or QNHC of hAWOs infected with a SARS-CoV-2 pseudovirus and SARS-CoV-2 virus significantly reduced viral replication and the number of infected cells in a dose-dependent manner (Han et al., 2020). Additionally, these drugs were able to inhibit SARS-CoV-2 pseudovirus infection in hAWOs transplanted in mice, suggesting their efficacy also in an *in vivo* model (Han et al., 2020). Overall, independent studies using hPSC-derived lung monolayer cultures and organoids confirmed the antiviral activity against SARS-CoV-2 *in vitro* of remdesivir, and to some extent camostat, and expanded the pool of candidate drugs to further pursue in clinical studies.

### 3.2 Cardiovascular system

Drug screening studies to identify treatments for COVID-19 have also adopted hPSC-based models of the cardiovascular system, including monolayer cultures of cardiomyocytes (hPSC-CM) (Bojkova et al., 2020, Garcia et al., 2020, Mills et al., 2020, Pérez-Bermejo et al., 2020, Samuel et al., 2020) and vascular organoids (Monteil et al., 2020). Cultures of hPSC-CM have been used to validate the antiviral activity of a selected protein kinase inhibitor identified from a drug screening assay based on Vero E6 cells (Garcia et al., 2020). In this study, Gracia et al. evaluated the efficacy of a chemical library comprising 430 kinase antagonists undergoing clinical testing and identified 34 hit drugs, all acting on DNA-Damage Response, ABL-24 BCR/MAPK, or mTOR-PI3K-AKT pathways. Among these candidates, berzosertib was selected for further investigation in hiPSC-CM. In agreement with the reduction of infected cells observed in Vero-E6 cells, berzosertib treatment decreased the levels of infectious virus present in the supernatant of infected hiPSC-CM cultures. Berzosertib treatment also reduced the number of apoptotic cells and restored hiPSC-CM contractility, increasing the number of beats per minute to levels comparable to controls (Garcia et al., 2020). Cultures of hPSC-CMs have also been adopted to test the efficacy of candidate drugs identified by literature search, such as E64d, Z-Phe-Tyr(tBu)-diazomethylketone (Z-FY-DK), CA-074, apilimod, bafilomycin, aprotinin and camostat (Pérez-Bermejo et al., 2020) or N-acetyl-L-leucyl-L-leucyl-Lmethionine (ALLM) and remdesivir (Bojkova et al., 2020). Treatment with the PIKfyve kinase inhibitor apilimod, the autophagy inhibitor bafilomycin and the viral RNA polymerase inhibitor remdesivir significantly reduced the number of infected cells. Treatment with cathepsin-B and -L inhibitors E64d and ALLM and cathepsin-L inhibitor Z-FY-DK also significantly decreased viral detection in infected cells while administration of cathepsin-B inhibitor CA-074 or TMPRSS2 inhibitors, such as aprotinin and camostat were ineffective, suggesting that in human cardiomyocytes SARS-CoV-2 uses cathepsin-L but not cathepsin-B or TMPRSS2 protease-mediated activation (Bojkova et al., 2020, Pérez-Bermejo et al., 2020). In another study, hPSC-CMs have been used to investigate the effects of drugs targeting proteins that mediate diastolic dysfunction induced by the “cytokine storm” also on SARS-CoV-2 viral replication (Mills et al., 2020). To identify candidate targets for pharmacological modulation, Mills et al. mimicked *in vitro* the cytokine storm induced by SARS-CoV-2 infection treating human cardiac organoids with combinations of inflammatory molecules. Analysis of inflamed cardiac organoids using phosphoproteomics in combination with single nuclei RNA-seq identified bromodomain protein 4 (BRD) as a promising target. Treatment of hPSC-CMs with BRD inhibitor INCB054329 at the same time of exposure to SARS-CoV-2 did not reduce viral replication or viral load while pre-treatment with INCB054329 before infection significantly reduced the viral load, decreased the number of infected cells and avoided sarcomere disorganisation, suggesting that BRD inhibitors are promising candidates for further investigation (Mills et al., 2020). Other studies have adopted the approach to reduce viral entry by either decreasing the expression of ACE2 and S (spike) priming proteases on host cells (Samuel et al., 2020) or targeting the virus directly using clinical-grade human recombinant soluble ACE2 (hrsACE2) (Bojkova et al., 2020, Monteil et al., 2020). To identify drugs able to decrease ACE2 expression, hESC-CMs were treated with FDA-approved drugs of the Selleckchem library and their ACE2 expression levels were monitored using high-throughput imaging (Samuel et al., 2020). To find additional compounds that could reduce ACE2 expression, the data obtained from this *in vitro* screening were used to train a deep learning model that was later applied for an *in silico* screening. The *in silico* screening identified several drugs able to reduce ACE2 expression that targeted proteins involved in androgen signaling. Lead drugs that block androgen signaling, such as dutasteride, spironolactone, camostat, ketoconazole and finasteride, significantly reduced the expression of ACE2 and TMPRSS2 in hESC-CMs. Additionally, preincubation with dutasteride significantly decreased entry of recombinant spike-RBD protein in hESC-CMs, while pretreatment with the androgen receptor agonist 5a-dihydrotestosterone significantly increased the internalization of spike-RBD protein. These findings suggest further exploration of drugs targeting androgen signaling as potential treatments for COVID-19 (Samuel et al., 2020). Using a different strategy, hPSC-CMs and capillary organoids have been used to test the potential use of clinical grade human recombinant soluble ACE2 (hrsACE2) to inhibit SARS-CoV-2 entry (Bojkova et al., 2020, Monteil et al., 2020). hrsACE2 has already been tested as a treatment for SARS-CoV-1 in clinical trials up to phase 2 (Khan et al., 2017). Treatment of hPSC-CMs with hrsACE2 significantly decreased spike protein expression in infected hPSC-CMs (Bojkova et al., 2020).. In agreement with this finding, vascular organoids infected with mixtures of SARS-CoV-2 and variable concentrations of hrsACE2 had significantly reduced levels of intracellular viral RNA (Monteil et al., 2020). The observed decrease in the amount of viral RNA was dose-dependent but incomplete even at the highest doses, suggesting that viral entry could be mediated by additional proteins or other mechanisms (Monteil et al., 2020). Nevertheless, the fact that hrsACE2 is able to significantly reduce SARS-CoV-2 cell entry during the early phases of the infection makes it a promising candidate treatment for COVID-19.

### 3.3 Kidney

The efficacy of hrsACE2 in reducing SARS-CoV-2 infection has also been validated in kidney organoids (Monteil et al., 2020). The observed efficacy of hrsACE2 in organoids modelling different organs encourages its further clinical investigation alone or in combination with other drugs.

### 3.4 Gastrointestinal tract

As the gastrointestinal tract is another target of SARS-CoV-2 infection, our group tested in hPSC-COs the antiviral efficacy of the three drugs identified as hit compounds in the hAWOs screening described previously (Han et al., 2020). Imatinib, MPA and QNHC were able to significantly reduced viral replication and the number of infected cells in a dose-dependent manner also in hPSC-COs infected with SARS-CoV-2 (Han et al., 2020), further suggesting their potential for future clinical trials. With a similar aim, Kruger et al. have used hPSC-derived intestinal organoids (PSC-HIOs) to evaluate the antiviral activity of three candidate drugs selected based on literature review (Krüger et al., 2020), including remdesivir, famotidine (Freedberg et al., 2020) and EK1 (Xia et al., 2020, Xia et al., 2019). Their results indicate that remdesivir and EK1 can significantly decrease the number of SARS-CoV-2 infected cells, while the histamine-2 antagonist famotidine was ineffective (Krüger et al., 2020). Overall, these studies seem to confirm the efficacy of remdesivir, imatinib, MPA and QNHC in inhibiting SARS-CoV-2 infection *in vitro* also in hPSC-based models of gastrointestinal tract and identify EK1 as a new promising drug for further evaluation.

### 3.5 Brain

Human cortical organoids have also been used in a drug repurposing study aimed to test the efficacy of sofosbuvir (Mesci et al., 2020). Sofosbuvir is an FDA-approved anti-hepatitis C drug that inhibits viral replication by binding to the RNA-dependent RNA polymerase active site (Elfiky, 2020a). Sofosbuvir is also effective against infections caused by other enveloped single-stranded, positive-sense RNA viruses, such as Zika virus, and it has been identified as a potential COVID-19 treatment by *in silico* studies (Elfiky, 2020b). Eight-week-old brain organoids treated with sofosbuvir after SARS-CoV-2 infection showed decreased viral accumulation and reduced cell death, as well as restored expression of the presynaptic protein vGLUT1, suggesting that sofosbuvir is a promising candidate to treat the neurological manifestations and damages caused by SARS-CoV-2 infection (Mesci et al., 2020).

## 4 Current limitations

While the hPSC-based platforms described here are useful human *in vitro* systems to study SARS-CoV-2 infection and identify candidate drugs, they are reductionist models that do not fully recapitulate every aspect of COVID-19 pathophysiology and should be interpreted with caution. Many hPSC-derived cell and organoid platforms are not able to generate all the cell types present in adult human organs. This is either because monolayer cultures and organoids are still immature compared to primary adult cells or because certain cell types elude the differentiation strategy. Ongoing efforts aimed at improving culture conditions are expanding the range of cell types that can be derived *in vitro* and the maturation stages that can be reached. The comparisons performed so far to benchmark hPSC-based platforms against primary human tissues support the notion that hPSC-derived cells and organoids are sufficiently mature to recapitulate several aspects of SARS-CoV-2 infection in different organs. However, primary human tissues and cellular models based on adult stem cells more accurately model certain features of COVID-19 pathophysiology, such as age-related responses to infection. Another limitation of the majority of hPSC-based platforms currently used in COVID-19 research is the lack of immune system components. Immune cells are emerging as crucial to many aspects of COVID-19 pathophysiology and disease outcome. The use of co-cultures with immune cells would enable one to study *in vitro* COVID-19 pathophysiology beyond the consequences of direct infection. However, even after the addition of immune cells, further work is needed to recapitulate the microenvironment and inter-organ communication that are present *in vivo*.

## 5 Concluding remarks

Notwithstanding these technical limitations, hPSC-based platforms have emerged as valuable tools to investigate several aspects of COVID-19 pathophysiology. As more studies will be performed, they will likely keep expanding the spectrum of tissues and cell types investigated and adopt more complex hPSC-based platforms. Co-cultures of tissue-specific hPSC-derived cells and organoids with hPSC-differentiated immune cells appear promising. These co-cultures would allow us to investigate the interactions between infected and immune cells, uncovering how immunomodulatory molecules released from infected cells affect immune components and how the immune response in turn impacts the infected tissue. They could also enable us to better evaluate how drugs are metabolized, increasing the faithfulness of *in vitro* drug screenings. To study the patient-specific responses to inflammation, an alternative to co-cultures with immune cells could be to treat tissue-specific cells and organoids with pro-inflammatory cytokines or anti-inflammatory treatments before or after infection. Additionally, since hPSC-derived cells and organoids are able to mimic different stages of fetal development, they can be useful to understand how maternal inflammatory responses might affect the growth of the fetuses and the effects of prenatal exposure to SARS-CoV-2. As additional receptors and cofactors that mediate SARS-CoV-2 entry have been recently discovered, their expression will probably be investigated in hPSC-based models. Likely, future investigations will also test several MOI in the same study and will monitor the long-term effects of SARS-CoV-2 infection and drug responses, expanding our understanding of infection progression and disease outcomes. We expect additional drug screening studies to compare the performance of the most promising drugs identified in different studies. Future drug screening studies could also more systematically identify molecules that worsen SARS-CoV-2 infection. Finally, hPSC-based platforms can be used to understand why the response to SARS-CoV-2 infection varies widely between individuals (Dobrindt et al., 2020) (Fig. 2). As hiPSCs can be generated from individuals with different genetic backgrounds and underlying medical conditions, collections of hiPSC lines may be used to complement on-going efforts investigating the genetic variants associated with susceptibility to infection and severity of COVID-19. HiPSCs-derived cells and organoids may further be employed in drug screening studies to evaluate the patient-specific responses to each drug, potentially helping to identify personalized therapies for COVID-19. Patient-specific iPSCs may also be helpful to assess drug safety in individuals with preexisting medical conditions, avoiding additional damage to compromised organs.

As more studies adopt and advance hPSC-based platforms to investigate COVID-19 pathophysiology, they will facilitate a better understanding of infection mechanisms and expedite the identification of candidate treatments, complementing the findings obtained from primary human tissues and animal models.

## Declaration of interests

The authors declare the following financial interests/personal relationships which may be considered as potential competing interests: Shuibing Chen reports financial support was provided by National Institute of Diabetes and Digestive and Kidney. Shuibing Chen reports financial support was provided by Bill and Melinda Gates Foundation.
